# Aberrant regional homogeneity in multiple frequency bands in patients with alcohol use disorder

**DOI:** 10.3389/fpsyt.2025.1549970

**Published:** 2025-11-05

**Authors:** Xia Ruan, Tingting Yu, Cixing You, Ming Yang, Jun Chen

**Affiliations:** ^1^ Department of Radiology, Qianjiang Central Hospital of Hubei Province, Qianjiang, Hubei, China; ^2^ Department of Radiology, Renmin Hospital of Wuhan University, Wuhan, Hubei, China

**Keywords:** alcohol use disorder, frequency band, regional homogeneity, resting-state functional magnetic resonance imaging, spontaneous brain activity

## Abstract

**Background:**

It is unclear whether the alterations in neural oscillations in patients with alcohol use disorder (AUD) are specific to different frequency bands. We applied the regional homogeneity (ReHo) approach to examine intrinsic functional connectivity variations in various frequency bands in AUD patients.

**Methods:**

Thirty-three AUD patients and 29 healthy controls (HCs) were enrolled in this study. The ReHo values in six frequency bands (conventional frequency band, 0.01–0.08 Hz; slow-2, 0.198–0.25 Hz; slow-3, 0.073–0.198 Hz; slow-4, 0.027–0.073 Hz; slow-5, 0.01–0.027 Hz; and slow-6, 0–0.01 Hz) were calculated and compared between the two groups. The performance of the ReHo on distinguishing AUD patients from HCs was examined using a receiver operating characteristic (ROC) curve. The correlation of functional changes in the network and alcohol dependence was evaluated.

**Results:**

Decreased ReHo values were detected in two frequency bands, and the areas of decreased ReHo values were mainly located in the left inferior parietal lobule (IPL) and right middle frontal gyrus (MFG); the diagnostic efficiency of the ReHo differences in brain areas was respectively 0.876 and 0.868, with sensitivities of 90.0% and 76.7% and specificities of 70.0% and 86.7%. The clinical scale scores were not significantly correlated with the ReHo values in specific brain areas.

**Conclusions:**

Our findings demonstrate that the widespread abnormal brain activity in AUD patients is characterized by distinct patterns of neural oscillatory power across multiple frequency bands. This exploration may provide an objective imaging basis for understanding the pathophysiological mechanisms of AUD.

## 1 Introduction

Alcohol is one of the most widely used addictive substances in the world, and drinking is closely related to a wide range of mental health issues, social problems, crime issues, and public health problems. There is no safe dose of alcohol, and long-term heavy drinking can lead to mild to moderate cognitive impairments, particularly in attention, executive functions, memory, visuospatial skills, motor abilities, and cognitive performance. It can also severely harm multiple organs in the human body and lead to increased cancer rates and mortality (1, 2). Therefore, using neuroimaging to understand the neurobiology of alcohol use disorder (AUD) and identifying appropriate therapeutic targets are becoming increasingly important. However, the exact mechanisms of AUD are not yet fully understood.

In recent years, a number of novel biomarkers for the diagnosis of AUD have been identified through brain imaging techniques. Resting-state functional magnetic resonance imaging (rs-fMRI) is a non-invasive method for quantifying the neural network structure and functional brain properties (3). The regional homogeneity (ReHo) method can be applied to analyze local signals in rs-fMRI, which is a highly reliable feature of the connectome of the human brain (4). Currently, ReHo analysis is widely used to assess physiological or pathological conditions to detect abnormalities in regional functional synchronization and to reveal the neural mechanisms of neurological and psychiatric disorders, such as headache disorders (5), schizophrenia (6), depression (7), and Alzheimer’s disease (8).

A majority of ReHo studies have examined conventional frequency bands (9, 10), but results from one frequency band lack frequency specificity. Conversely, rare studies have explored the local characteristics of the blood oxygen level dependent (BOLD) signal in different frequency bands, even though they may offer useful information. Different oscillation frequencies could be used to reflect various aspects of brain function (11, 12). The study of Baria et al. (13) concluded that resting-state BOLD oscillations exhibit frequency-dependent anatomically constrained spatial structure in the human brain throughout the full BOLD bandwidth. Liu et al. (14) found that a frequency-dependent genetic modulation of spontaneous neuronal activity, which may support spontaneous neural activity across different frequency bands, may be driven by different genetic underpinnings. A previous study by Song et al. (15) has shown that the frequency-specific ReHo variations across brain regions may originate from their distinct cytoarchitectonic organization or synaptic diversity. Therefore, distinct frequency-specific ReHo characteristics could be more sensitive markers for different tasks or pathophysiological states, advancing the understanding of the neural–physiological basis of regional structural–functional specificity and its link to regional homogeneity. Typically, the power spectrum has five different frequency bands (16): slow-2, slow-3, slow-4, slow-5, and slow-6. Most studies have ignored the very low-frequency band (slow-6, <0.01 Hz), but slow-6 has been shown to be significant in physiological and pathological investigations (17–19). Many studies have investigated the effects of frequency band differences on functional brain connectivity, such as obsessive compulsive disorder (20), schizophrenia (21), acute pericoronitis (22), and tension-type headache (23). However, it is unclear how ReHo changes with frequency in patients with AUD.

Therefore, we performed rs-fMRI to detect the frequency-dependent effects of ReHo in patients with AUD and then correlated these changes with clinical and neuropsychological data. We tested the ability of ReHo analysis to distinguish between AUD and healthy controls (HCs) using a receiver operating characteristic (ROC) curve.

## 2 Materials and methods

### 2.1 Subjects

The study was approved by the Medical Ethics Committee at Renmin Hospital of Wuhan University and followed the principles of the Declaration of Helsinki. Written informed consent was obtained from all subjects.

Thirty right-handed male AUD patients and 30 HCs were recruited from the primary care outpatient department of Renmin Hospital of Wuhan University, and they were matched on age, sex, education, and handedness.

AUD patients must match the diagnostic criteria for AUD in the American Psychiatric Association’s Diagnostic and Statistical Manual of Mental Disorders, Fifth Edition (DSM-5). The inclusion criteria of AUD patients were as follows: a history of alcohol dependence for no less than 10 years, Alcohol Dependence Scale (ADS) score ≥14 and Michigan Alcohol Screening Test (MAST) score ≥ 6 (24), and no prior treatment history for AUD. HCs were those who had never or very seldom consumed alcohol (<1 standard unit per time) (25).

Subjects were excluded due to any of the following: 1) an individual who exhibits psychotic symptoms or whose first-degree relative has been diagnosed with psychosis; 2) history of addiction to substances other than alcohol; 3) with organic brain disease or severe physical disease; 4) a history of cranial trauma, cranial surgery, brain tumor, and coma; 5) people with previous seizures or a family history of epilepsy; 6) patients who have been treated with antipsychotic medication or who are receiving medication; and 7) claustrophobia or any MRI contraindications.

The time interval between the last alcohol consumption and MR examination was 3 weeks for all subjects in this study to exclude the effects of acute alcohol intake.

### 2.2 Cognitive and alcohol level evaluation

Before undergoing MRI scans, all subjects were administered the Mini-Mental State Examination (MMSE) and Montreal Cognitive Assessment (MoCA); to evaluate the level of alcohol dependence, only ADS and MAST scores from the AUD group were collected.

### 2.3 MRI acquisition

MRI scans were acquired using a 3.0-T MRI scanner (Discovery 750W Silent MR, GE Healthcare, Milwaukee, WI, USA). To avoid subject head movement artifacts during the experiment, the matching rubber soft plugs were used to fix the head. To reduce equipment noise, soft foam earplugs were also used. All subjects were asked to be quiet and relaxed, remain awake, keep their eyes closed, and think of nothing in particular. Participants with lesions of the brain were excluded from T2-weighted imaging (T2WI) and T2-Fluid Attenuated Inversion Recovery (T2-FLAIR) images. High‐resolution structural data were obtained in sagittal position: repetition time (TR) = 8.5 ms, echo time (TE) = 3.3 ms, field of view (FOV) = 240 mm × 240 mm, flip angle (FA) = 12°, matrix size = 256 × 256, slice thickness = 1.0 mm, no slice gap, and voxel size = 1 mm × 1 mm × 1 mm. The scanning parameters of fMRI data were as follows: TR = 2,000 ms, TE = 25 ms, FOV = 240 mm × 240 mm, FA = 90°, slice thickness = 3.5 mm, no slice gap, matrix size = 64 × 64, interleaved axial slices = 40, and volumes = 240. The visual inspection of all MR images was carried out to make sure that none of the images included in the study had visible artifacts.

### 2.4 MRI data preprocessing and analysis

#### 2.4.1 Data preprocessing

MRI data were preprocessed using the Statistical Parametric Mapping software (SPM12) (http://www.fil.ion.ucl.ac.uk/spm) and Resting-State fMRI Data Analysis Toolkit plus (RESTplus) (http://restfmri.net/forum/RESTplus). The main preprocessing steps were as follows: 1) remove the first 10 time points because of inhomogeneities in the magnetic field and subjects’ maladaptive interferences with the environment; 2) slice timing; 3) realign, subjects with head movement >2.0 mm or 2.0° were excluded; 4) normalizing the data to the Montreal Neurological Institute (MNI) space via the DARTEL technique (26); 5) detrend; 6) nuisance covariate regression, including the Friston 24-parameter model, white matter, and cerebrospinal fluid signals; and 7) filter (conventional frequency band, slow-2 band, slow-3 band, slow-4 band, slow-5 band, and slow-6 band).

#### 2.4.2 ReHo calculation

For filtered data in each band, the ReHo value was calculated using Kendall’s coefficient of concordance of the time series of every 27 nearest neighboring voxels. For standardization, the ReHo z-score map for each subject was obtained using Fisher’s r-to-z transformation. Spatial smoothing was performed using a 6 mm × 6 mm × 6 mm Gaussian kernel after ReHo calculation.

### 2.5 Statistical analysis

#### 2.5.1 Clinical information

Baseline clinical information was compared between the two groups using the Statistical Package for the Social Sciences (SPSS) version 26.0 software (IBM, Armonk, NY, USA). Demographic and clinical data for both groups were tested using the Shapiro–Wilk test and independent samples *t*-tests. The threshold was set at a significant level of *p* < 0.05.

#### 2.5.2 ReHo analysis

Group comparisons in ReHo were conducted using two-sample *t*-tests in the SPM12 software. Age, education level, and mean framewise displacement were used as covariates in two-sample *t*-tests. ReHo differences in six different frequency bands were analyzed, and regions where the differences were significant were pinpointed. Multiple comparisons were corrected using a cluster-level family-wise error (FWE) method, resulting in a cluster-defining threshold of *p* < 0.001 and a corrected cluster significance of *p* < 0.05. Crucially, the minimum cluster size was independently determined for each frequency band (conventional frequency band: cluster size = 85, slow-3 band: cluster size = 126) through separate Monte Carlo simulations. This approach accounts for intrinsic differences in spatial smoothness and data structure between bands, ensuring valid inference specific to each band’s characteristics.

#### 2.5.3 ROC curve and correlation analysis

To determine if the ReHo values may be used as a biological marker for differentiating AUD from HCs, the ReHo values from regions with significant differences between groups were extracted for the ROC curve.

To investigate the specific relationships between ReHo values in different frequency bands and MAST/ADS scores, we performed a correlation study using the SPSS version 26.0 software. We extracted ReHo values from regions with significant differences between groups and analyzed the relationships between ReHo values and the patients’ MAST/ADS scores. We calculated Spearman’s correlation coefficient.

## 3 Results

### 3.1 Clinical information

No participants were excluded following correction for head motion. Finally, 30 AUD patients and 30 HCs were included in this research. The clinical information of the two groups is shown in **Table 1**. In terms of gender, age, education, handedness, and mean Framewise Displacement (FD) , no statistically significant difference was found. Nonetheless, the MMSE and MoCA scores of the AUD group were considerably lower (*p* < 0.05) than those of the HC group.

**Table 1:** Demographic and clinical characteristics.

Characteristics	AUD (n = 30)	HCs (n = 30)	*P*-Value
Age (years)	53.03 ± 5.49	50.13 ± 5.89	0.053
Gender (male/female)	30/0	30/0	–
Education level (years)	11.17 ± 2.78	10.73 ± 3.12	0.572
Handedness (R/L)	30/0	30/0	–
MoCA	25.17 ± 2.83	26.63 ± 1.88	0.021
MMSE	26.73 ± 1.86	28.00 ± 1.39	0.004
MAST	8.83 ± 3.36	–	–
ADS	17.03 ± 3.54	–	–
Mean framewise displacement	0.087 ± 0.038	0.086 ± 0.069	0.917

Values are expressed as means ± standard deviations or frequencies. *p* < 0.05 was considered statistically significant.

AUD, alcohol use disorder; HCs, healthy controls; MoCA, Montreal Cognitive Assessment; MMSE, Mini-Mental State Examination; MAST, Michigan Alcohol Screening Test; ADS, Alcohol Dependence Scale.

### 3.2 ReHo analysis

**Figure 1:**
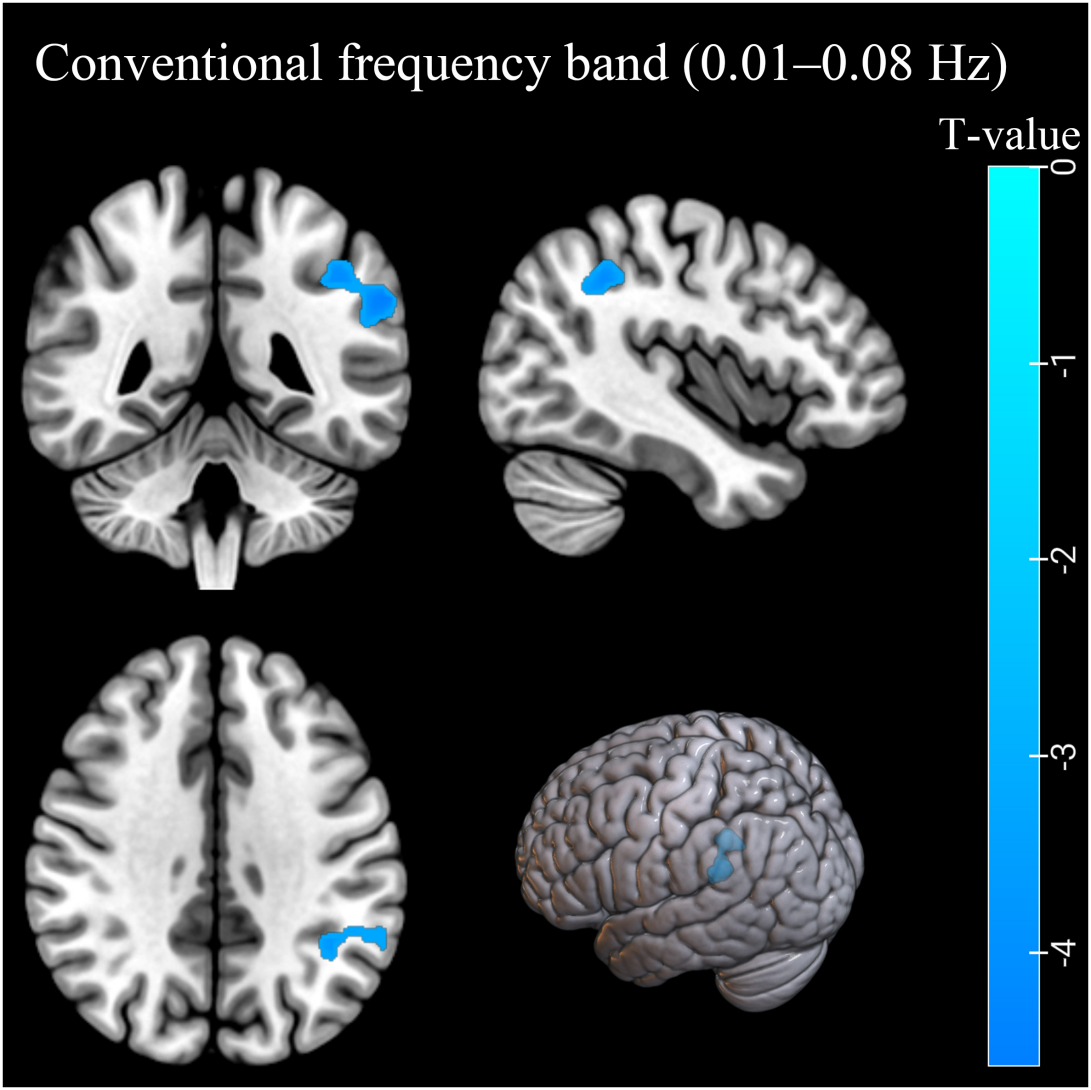
The ReHo differences in the conventional band (0.01–0.08 Hz) between AUD patients and HCs. Blue denotes lower ReHo values in AUD patients compared to HCs. ReHo, regional homogeneity; AUD, alcohol use disorder; HCs, healthy controls.

**Figure 2:**
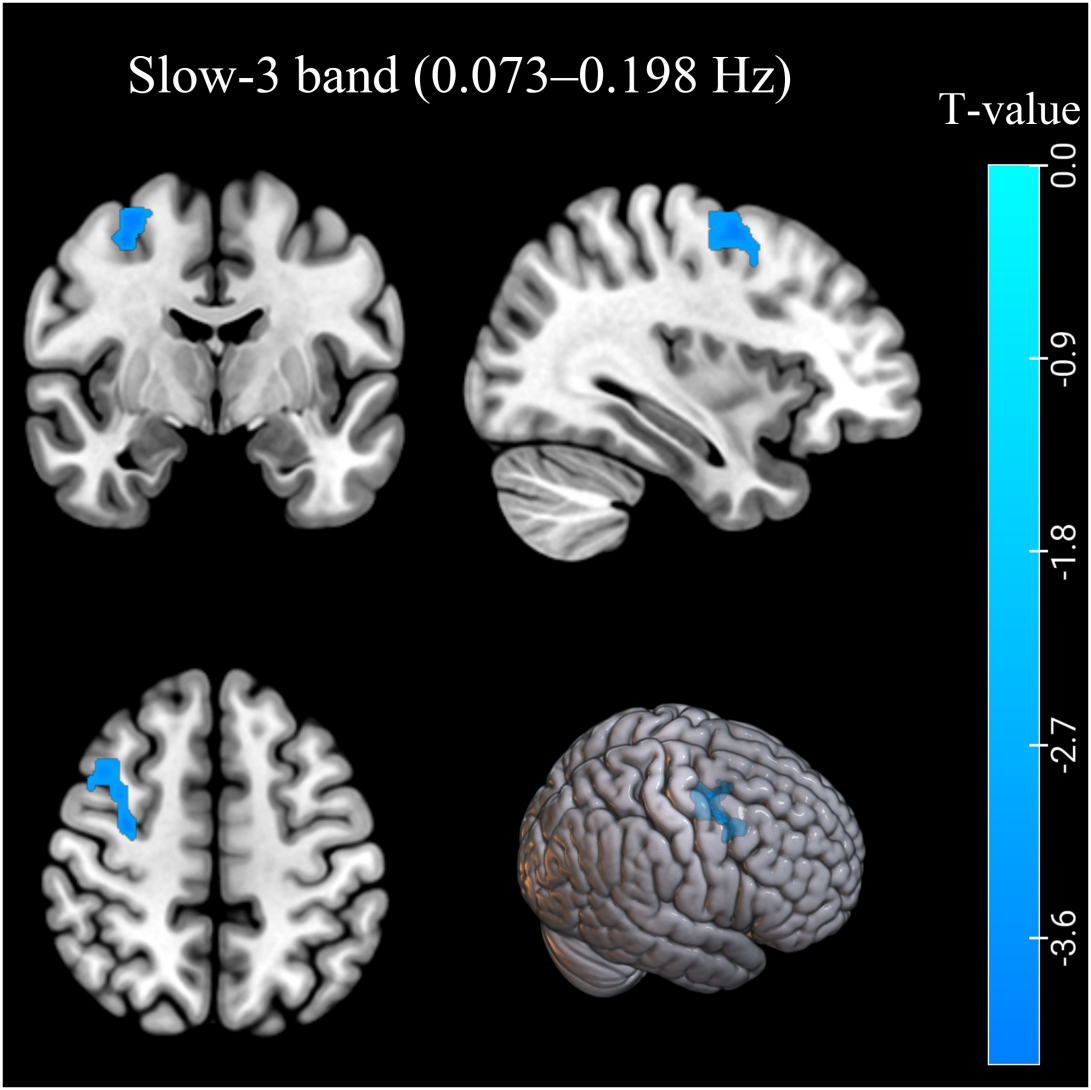
The ReHo differences in the slow-3 band (0.073–0.198 Hz) between AUD patients and HCs. Blue denotes lower ReHo values in AUD patients compared to HCs. ReHo, regional homogeneity; AUD, alcohol use disorder; HCs, healthy controls.

In the conventional frequency band, the ReHo values decreased in the left inferior parietal lobule (IPL). The ReHo values in the right middle frontal gyrus (MFG) were decreased in the AUD group in the slow-3 band (**Table 2**; **Figures 1**, **2**). The ReHo values were not significantly different in slow-2, slow-4, slow-5, and slow-6 bands.

**Table 2:** The ReHo difference in each frequency band between patients with AUD and HCs (*p* < 0.05, cluster-level FWE corrected).

Brain regions	BA	Cluster size	Peak MNI coordinates			T value
			X	Y	Z	
Conventional frequency band (0.01–0.08 Hz)						
L-Inferior parietal lobule	40	85	−57	−48	30	−4.6308
Slow-3 band (0.073–0.198 Hz)						
R-Middle frontal gyrus	6	126	33	−3	54	−4.2819

ReHo, regional homogeneity; MNI, Montreal Neurological Institute; BA, Brodmann area; AUD, alcohol use disorder; HCs, healthy controls; L, left; R, right; FWE, family-wise error.

### 3.3 ROC curve

**Figure 3:**
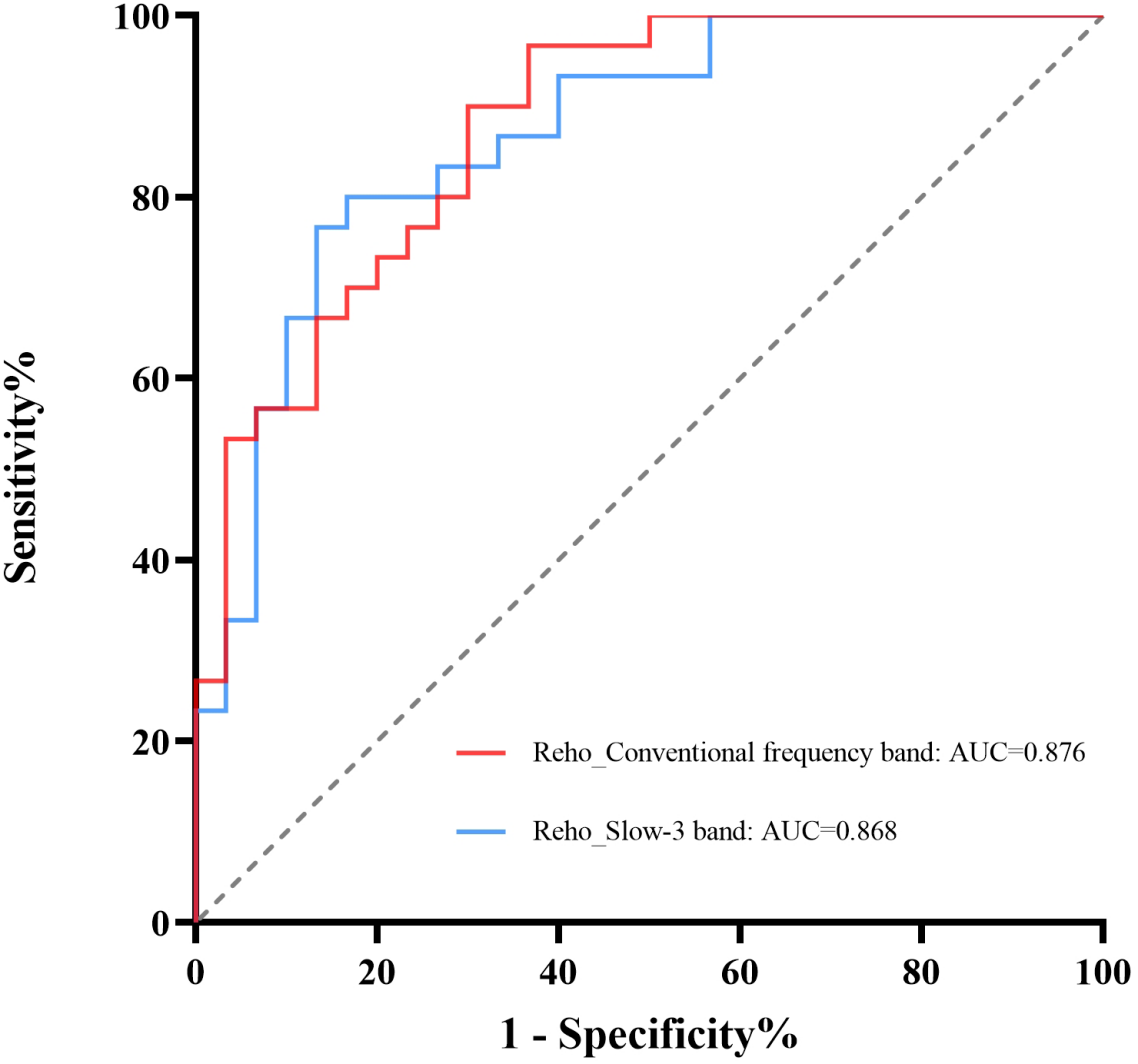
ROC curve analysis of ReHo values from regions with significant differences. The diagnostic efficiency of the ReHo differences in brain areas ranged from 0.876 to 0.868, with sensitivities of 76.7%–90.0% and specificities of 70.0%–86.7%. ReHo, regional homogeneity; ROC, receiver operating characteristic.

According to our research, the ROC curve showed good AUC values (range 0.868–0.876) for ReHo values in various bands. Further diagnostic study found that the ReHo values can distinguish the AUD patients from the HCs with good sensitivities (range 76.7%–90.0%) and specificities (range 70.0%–86.7%) (**Table 3**; **Figure 3**).

**Table 3:** ROC curve for ReHo differences in brain areas between AUD patients and HCs.

Brain area	AUC	Sensitivity, %	Specificity, %	Accuracy
L-IPL (conventional frequency band)	0.876	90.0%	70.0%	0.820
R-MFG (slow-3 band)	0.868	76.7%	86.7%	0.836

ROC, receiver operating characteristic; AUC, area under the curve; ReHo, regional homogeneity; AUD, alcohol use disorder; HCs, healthy controls; IPL, inferior parietal lobule; MFG, Middle frontal gyrus; L, left; R, right.

### 3.4 Correlation analysis

**Figure 4:**
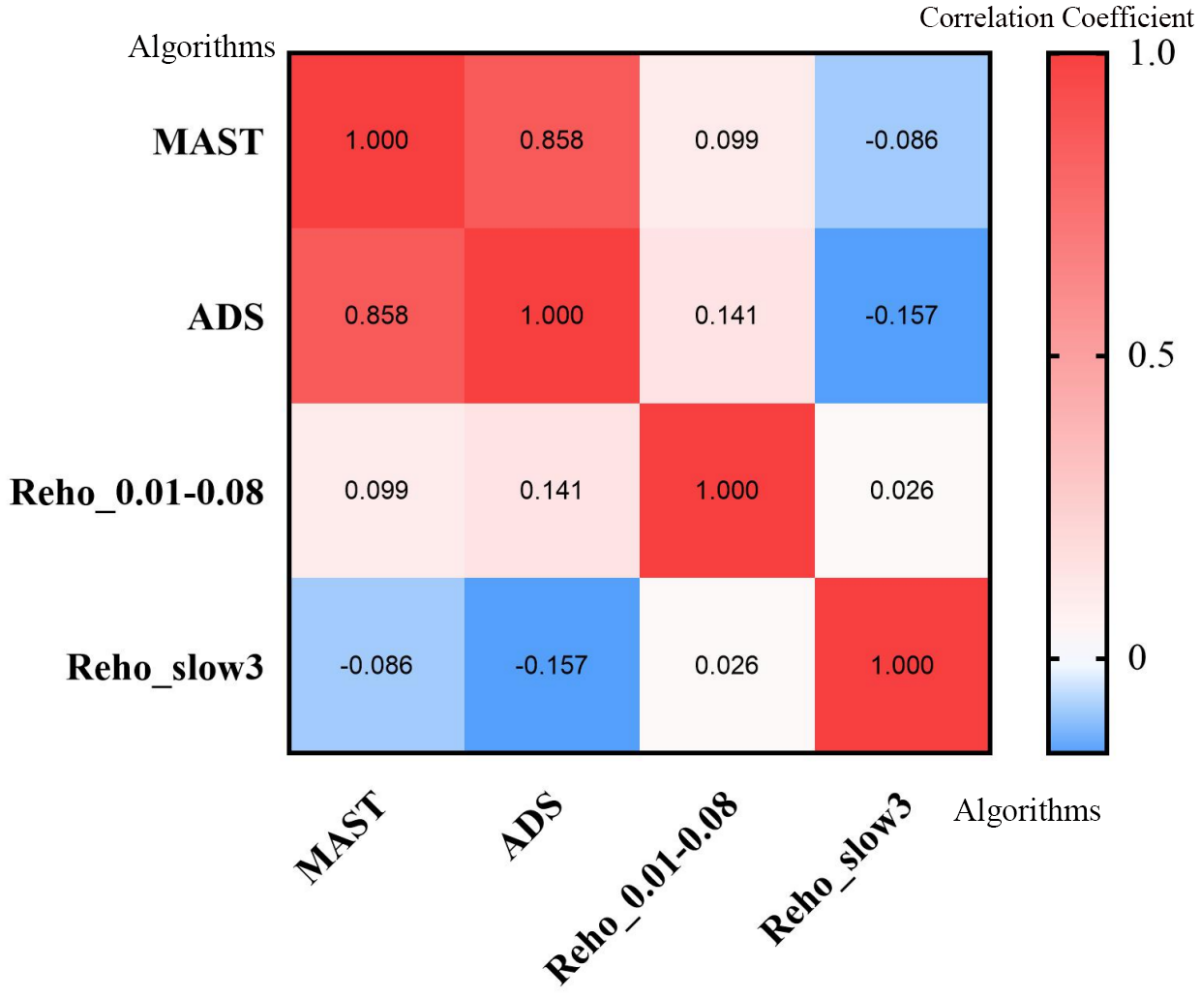
Heatmap of correlation analysis between MAST score, ADS score, and ReHo values from regions with significant differences in different frequencies. ReHo, regional homogeneity; MAST, Michigan Alcohol Screening Test; ADS, Alcohol Dependence Scale.

The MAST or ADS scores were not significantly correlated with the ReHo values from regions with significant differences between groups (**Figure 4**).

## 4 Discussion

ReHo is a method to examine local signal variation in brain activity (4). Many studies have indicated that the low-frequency fluctuation of fMRI was related to spontaneous neuronal activity in certain areas of the brain (27, 28). Currently, fMRI studies on AUD patients have mostly focused on the conventional frequency band (9, 10). Previous results (29) have shown that brain activity in AUD patients may be different in multiple frequency bands, but no studies have explored the regional homogeneity of multiple frequency bands in AUD patients. Therefore, this study investigated the brain activity of AUD patients using the ReHo method in six different frequency bands.

Significant variations in ReHo values were seen in several regions in the two frequency bands. These findings suggested frequency-dependent effects of altered brain activity in AUD patients.

In this study, the AUD group exhibited decreased ReHo values in the left IPL (BA40) in the conventional frequency band and the right MFG (BA6) in the slow-3 band. In contrast, ReHo values in slow-2, slow-4, and slow-5 bands showed no significant group difference. The decreased ReHo values of the IPL (BA40) and MFG (BA6) indicated decreased local synchronization of BOLD signal fluctuations.

The MFG is an important part of the frontal lobe, which interacts with several brain regions to accomplish many higher brain activities, such as processing various sensory information, cognition, error processing, impulse control, and decision-making processes. The current studies (30–33) have shown that alcohol abuse can lead not only to reduced MFG activation but also to changes in volume. Quaglieri et al. (34) found clusters of deactivation in the MFG of patients with AUD, and their findings suggest that patients with AUD have difficulty adapting to the changing contingencies of reward-oriented decision-making. The MFG is also a key part of language processing and has functions of coordinating different information, so the damage to this brain region was related to reduced language function in AUD patients (35). Based on these findings, our results of the reduction in regional homogeneity in the MFG are consistent with those of previous studies on alcohol abuse (30, 31, 34). Therefore, the change of MFG function may be one of the main reasons for losing control of alcohol dependence behavior.

The IPL is involved in attention allocation and the assessment of decisions (36, 37). A follow-up study of short-term abstinent alcoholics by Camchong et al. found that compared with abstainers, relapsers showed significantly decreased resting-state synchrony in the IPL (36). The results of this study suggest that relapsers have lower organization in the attention network, a process required for proper decision-making and evaluation, such as deciding to ingest suppressive drugs to avoid relapse. Previous studies have also shown that alcohol-induced differences in degree centrality are mainly located in the IPL and that functional connectivity between the bilateral IPL may be related to alcohol dependence and cognitive impairment (38). Thus, the reduced ReHo of the IPL in this study may be one of the reasons for impaired attention and decision-making function in AUD patients.

The observed ReHo reductions in the MFG and IPL may reflect disruptions in large-scale intrinsic connectivity networks critical for cognitive and behavioral control. Recent studies have demonstrated that aberrant functional integration within the frontoparietal network (FPN) contributes to impaired decision-making and attentional deficits (39–41). Specifically, the MFG (a key node of the FPN) and the IPL (part of both the FPN and dorsal attention network) are hubs for top-down regulation of goal-directed behavior (42, 43). Alcohol-related ReHo reductions in these regions may indicate localized desynchronization, which could propagate to network-level dysfunctions, including disrupted communication between the FPN and default mode network (DMN) (44). Such network disorganization aligns with the loss of inhibitory control and compulsive alcohol use in AUD patients.

We found ReHo differences in two bands, but not in slow-2, slow-4, slow-5, and slow-6 bands. Slow-2 and slow-6 mainly reflect very physiological noise and low-frequency drift, which is probably the main reason (45). There were no significant ReHo differences in these frequency bands that are most susceptible to non-neural signals, indicating that the observed ReHo abnormalities in other frequency bands were unlikely to be caused by such artifacts, but were more likely to reflect the actual neural changes in the AUD. This further supports the finding of this study that the frequency band specificity has a neural significance. Although slow-4 and slow-5 bands are the two main frequency ranges in which gray matter-related oscillations occur at resting state (16), this study did not find significant differences in the ReHo of the AUD and HC groups in these two frequency bands. This indicates that the network abnormalities in AUD patients may not be dependent on the local synchrony changes in the traditional gray matter frequency bands but are transmitted through conventional frequency bands, the slow-3 frequency band, or cross-frequency band coupling (such as phase–amplitude coupling) (46, 47). The decreased ReHo in the differential frequency bands of AUD patients may indicate the desynchronization of local sub-networks (such as FPN nodes), but by retaining the integration ability of slow-4 and slow-5, the basic network architecture can be maintained.

Furthermore, the potential diagnostic value of our findings was explored. The ROC curve showed good AUC values for ReHo values from regions with significant differences in various bands. Further diagnostic study found that the ReHo values can distinguish the AUD patients from the HCs with good sensitivities and specificities. In addition, this study did not show any association between MAST/ADS scores and the ReHo values from the regions with significant differences in the conventional and slow-3 bands in AUD patients.

There were some limitations in our study. First, the sample size was relatively small, although rs-fMRI studies with more than 16 subjects per group are considered acceptable (48). Future replication in larger cohorts is essential. Second, the results may not apply to female AUD subjects due to the fact that only men were included. Finally, this study was cross-sectional, and dynamic changes of ReHo in AUD patients could not be observed at various stages. Further longitudinal studies would be beneficial in addressing this issue.

## 5 Conclusion

The ReHo analysis demonstrated aberrant signal fluctuations in certain areas of the brains of AUD patients. Furthermore, altered spontaneous brain activity was frequency-specific. The use of a particular frequency band may be helpful in measuring the intrinsic activity of AUD patients. Our results will help to better understand the neuropathological mechanism of AUD, which can provide a foundation for further exploration.

## Author’s note

This research is related to a previous study by the same authors titled “A voxel-level resting-state fMRI study on patients with alcohol use disorders based on a power spectrum slope analysis method”. The previous study was performed on whether PSS, as a novel method, can well detect abnormal local brain activity in AUD patients and the current submission is focusing on aberrant regional homogeneity in multiple frequency bands. This article follows the methodology explained in 4, 16.

## Data Availability

The raw data supporting the conclusions of this article will be made available by the authors, without undue reservation.
